# Superdormant Spores as a Hurdle for Gentle Germination-Inactivation Based Spore Control Strategies

**DOI:** 10.3389/fmicb.2018.03163

**Published:** 2019-01-04

**Authors:** Yifan Zhang, Alexander Mathys

**Affiliations:** Sustainable Food Processing Laboratory, Institute of Food, Nutrition and Health, Department of Health Science and Technology, ETH Zurich, Zurich, Switzerland

**Keywords:** bacterial spore, superdormant, germination, inactivation, isolation, characterization, mechanism, gentle spore control

## Abstract

Bacterial spore control strategies based on the germination-inactivation principle can lower the thermal load needed to inactivate bacterial spores and thus preserve food quality better. However, the success of this strategy highly depends on the germination of spores, and a subpopulation of spores that fail to germinate or germinate extremely slowly hinders the application of this strategy. This subpopulation of spores is termed ‘superdormant (SD) spores.’ Depending on the source of the germination stimulus, SD spores are categorized as nutrient-SD spores, Ca^2+^-dipicolinic acid SD spores, dodecylamine-SD spores, and high pressure SD spores. In recent decades, research has been done to isolate these different groups of SD spores and unravel the cause of their germination deficiency as well as their germination capacities. This review summarizes the challenges caused by SD spores, their isolation and characterization, the underlying mechanisms of their germination deficiency, and the future research directions needed to tackle this topic in further depth.

## Introduction

Bacterial spores are widely distributed and can cause spoilage and food-borne diseases, leading to economic losses and endanger public health (Setlow et al., 2012; Banawas et al., 2013). They are extremely resistant to heat, dehydration, and chemical or physical stresses, making them the main challenge of sterilization processes (Setlow, 2006, 2007; Setlow and Johnson, 2007; Patrignani and Lanciotti, 2016; Zhang et al., 2018). Because of their resistance, intensive wet heat treatment, generally at a temperature higher than 100°C, is usually applied to inactivate spores in food products (Storz and Hengge, 2010; Georget et al., 2013), and such processing procedures often cause an unwanted loss of food quality (Sevenich and Mathys, 2018). Therefore, development of effective gentle non-thermal spore decontamination strategies is currently of high interest (Storz and Hengge, 2010; Zhang et al., 2018).

**FIGURE 1:**
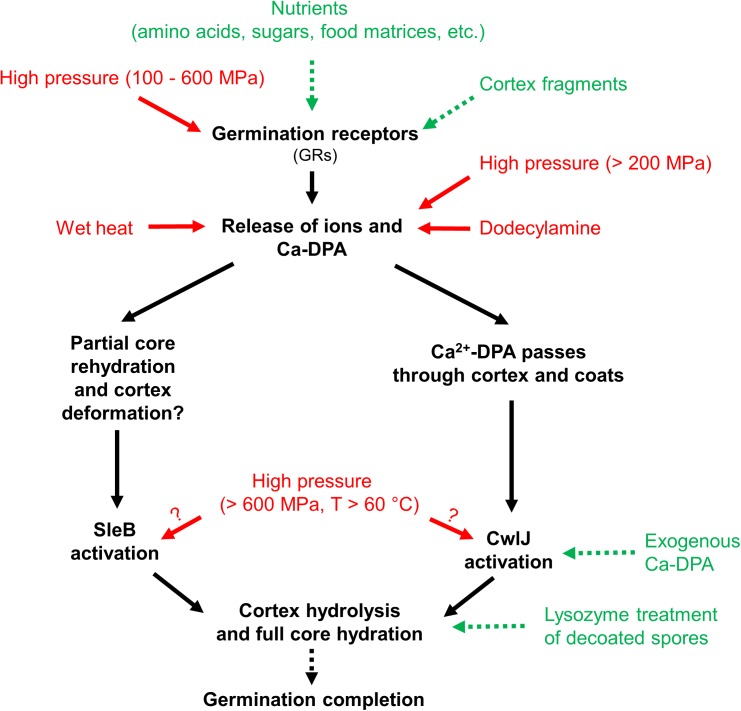
Overview of germination stimuli and proposed germination pathways of *Bacillus subtilis* spores. Stimuli that lead to germination are shown as green with dashed arrows and stimuli that lead to germination and possible inactivation are shown as red with solid arrows. Graph modified from Reineke et al. (2013), with permission from Elsevier.

Research has revealed that spores lose their extreme resistance after germination and become easier to kill, e.g., by milder heat inactivation (Collado et al., 2004; Setlow, 2006; Abee et al., 2011; Lovdal et al., 2011). Moreover, spore germination can be artificially triggered by nutrient germinants (van der Voort et al., 2010; Baier et al., 2011; Sevenich and Mathys, 2018), as well as non-nutrient stimuli, e.g., Ca^2+^-dipicolinic acid (Ca^2+^-DPA), and isostatic high pressure (HP) (Gould, 1970, 2006; Baier et al., 2011; Reineke et al., 2013). The overview of germination stimuli and proposed germination pathways for *Bacillus subtilis* spores is shown in Figure 1. Based on this overview, gentle spore control strategies could be developed to achieve spore decontamination without largely compromising the food quality at the same time. For example, so-called “germination-inactivation” methods that first artificially trigger the germination of spores, and then eliminate those spores which lost their extreme resistance during germination with a mild inactivation step (Gould, 2006; Lovdal et al., 2011; Nerandzic and Donskey, 2013).

However, the germination behavior of spores is highly heterogeneous (Chen et al., 2006; Gould, 2006; Indest et al., 2009; Eijlander et al., 2011; Stringer et al., 2011; Setlow et al., 2012). Most spores can germinate rapidly after being exposed to germinant stimuli, but a subpopulation referred to as superdormant (SD) spores remained dormant or germinated extremely slowly (Gould, 2006; Ghosh and Setlow, 2009; Zhang et al., 2010; Rodriguez-Palacios and LeJeune, 2011; Sevenich and Mathys, 2018). These SD spores are the major limitations of the germination-inactivation spore control strategy. With the increased awareness of the importance of this subpopulation, more research has progressively shifted their focus to better understand this subpopulation, either in aggregate or at single cell level (Davey and Kell, 1996; Margosch et al., 2004; Ghosh and Setlow, 2009; Eijlander et al., 2011; Kong et al., 2011; Wang et al., 2011; Zhang et al., 2012; Perez-Valdespino et al., 2013). This review summarizes the challenges that SD spores cause, their isolation and characterization, the mechanisms of their superdormancy, and potential future research directions.

## Challenges Associated With Sd Spores

Owing to their germination deficiency, SD spores are considered to be the main obstacle to the effective application of germination-inactivation spore control methods (Ghosh and Setlow, 2009; Lovdal et al., 2011; Wang et al., 2012; Markland et al., 2013a; Olguin-Araneda et al., 2015). For example, the tyndallization strategy is based on a germination-inactivation concept (Tyndall, 1877), and is considered to not be fully reliable due to the presence of superdormant spores (Gould et al., 1968; Gould, 2006).

Additionally, the presence of SD spores complicates spore quantification and presents potential limitations for the reliability of challenge and sterilization tests. They may stay dormant and remain undetectable during recovery, but germinate later and proliferate, causing spoilage or even foodborne diseases (Deng et al., 2015; Silvestri et al., 2015). For example, spores formed by some *Clostridium* species could recover from superdormancy during long-term storage and become viable afterward, posing a potential risk (Esty and Meyer, 1922; Deng et al., 2015, 2017).

Moreover, the presence of SD spores also complicates decisions regarding the duration of antibiotic treatment for *Bacillus anthracis* infection. A number of antibiotics can destroy germinated *B. anthracis* spores, but SD spores can remain unaffected. Therefore, the ability of SD spores to stay in a dormant state and germinate after antibiotic treatment is stopped makes them capable of causing fatal diseases (Brookmeyer et al., 2003; Heine et al., 2007; Setlow et al., 2012).

Furthermore, SD spores were found to be much more resistant than the overall spore population (Ghosh et al., 2009; Markland, 2011; Rodriguez-Palacios and LeJeune, 2011; Markland et al., 2013b). For example, isolated *Bacillus* nutrient-SD spores had increased heat resistances as compared to the initial spore population (Ghosh et al., 2009). This indicates SD spores might be the main contribution to the log_10_ non-linear tailing phenomenon of spore inactivation kinetic curves, which complicates the prediction and modeling of spore behavior (Eijlander et al., 2011; Doona et al., 2012, 2016b,c, 2017; Sevenich and Mathys, 2018). Furthermore, their above-average resistance is a clear concern for the food industry, since the treatment intensity that inactivates the majority of the population might not be able to inactivate SD spores, leading to insufficient sterilization.

## SD Spore Isolation and Characterization

Superdormant is a relative term and it describes a subpopulation of spores that is phenotypically different on their germination capacity compared to the rest of the population. Notably, it is not a static subpopulation of spores but rather a subpopulation that depends largely on the germination/isolation conditions and the cut-off point, e.g., germination trigger intensity and maximum treatment time, defined by the researchers. However, the subpopulation that fails to germinate after intensive germination stimuli is generally referred as SD spores (Ghosh and Setlow, 2009). SD spores are grouped into different categories according to their germination stimuli, e.g., nutrient-SD spores, Ca^2+^-dipicolinic acid SD (Ca^2+^DPA-SD) spores, dodecylamine-SD spores, and high pressure superdormant (HPSD) spores. Buoyant density centrifugation method was used to isolate nutrient-SD spores. The main principle of this method is that dormant spores have higher wet densities thus would pellet during centrifugation in a density gradient medium such as Nycodenz^®^. The germinated spores, which have significantly lower densities, would float (Ghosh and Setlow, 2009). This method was shown to effectively isolate Ca^2+^DPA-SD and dodecylamine-SD spores as well (Ghosh and Setlow, 2010; Perez-Valdespino et al., 2013). Additionally, new tools have been developed to characterize SD spores. These include Raman spectroscopy, differential interference contrast and phase-contrast microscopy (Zhang et al., 2010; Kong et al., 2011), and tracking of single cell germination/outgrowth using microtiter plates containing one spore per well (Webb et al., 2007; Wells-Bennik et al., 2016). Details, additional methods and tools to study spore heterogeneity were summarized by Setlow et al. (2012) and Wells-Bennik et al. (2016).

In addition to the isolation and characterization of SD spores, the mechanisms underlying their germination deficiency have also been investigated (Ghosh and Setlow, 2010). So far, nutrient-SD spores have been studied extensively, while Ca^2+^DPA-SD spores and dodecylamine-SD spores have been characterized to a limited extent, but HPSD spores have not yet been isolated and studied for their properties. More information can be seen in Table 1.

**Table 1:** Percentage of superdormant (SD) spores in various isolation conditions and proposed superdormancy mechanisms.

SD spore type	Germination stimulus	Species	ca. % SD spores	Proposed superdormancy mechanisms	Reference
	Valine (10 mM)	*B. subtilis*	1.1		Chen et al., 2014
	Valine (10 mM)	*B. subtilis*	4		Zhang et al., 2012^1^
	Valine (10 mM)	*B. subtilis*	3.8		Ghosh and Setlow, 2009
	Valine (300 μM)	*B. subtilis*	58		Ghosh and Setlow, 2009
	10 × LB medium^2^	*B. subtilis*	0.7		Ghosh and Setlow, 2009
	AGFK^3^	*B. subtilis*	12	Permanent cause: lower GR	Ghosh and Setlow, 2009
	AGFK^4^	*B. subtilis*	6	levels Transient cause:	Zhang et al., 2012^1^
Nutrient-SD	Glucose (10 mM)	*B. megaterium*	3.5	activation status	Ghosh and Setlow, 2009
	Glucose (200 μM)	*B. megaterium*	38	(Ghosh et al., 2009, 2012;	Ghosh and Setlow, 2009
	10 × LB medium	*B. megaterium*	0.5	Wei et al., 2010;	Ghosh and Setlow, 2009
	Alanine (50 mM)	*B. cereus*	5.3	Zhang et al., 2012)	Ghosh and Setlow, 2010
	Inosine (5 mM)	*B. cereus*	2.3		Ghosh and Setlow, 2010
	Inosine (250 μM)	*B. cereus*	12		Ghosh and Setlow, 2010
	Inosine (5 mM, no heat activation)	*B. cereus*	12		Ghosh and Setlow, 2010
Ca^2+^DPA-SD	Ca^2+^-DPA (60 mM)	*B. subtilis*	0.9 (0.5–1.6)	Coat defect, low levels of CLE CwlJ	Perez-Valdespino et al., 2013
Dodecylamine-SD	Dodecylamine (1.2 mM)	*B. subtilis*	0.4 (0.1–1.1)	Not clear	Perez-Valdespino et al., 2013
High pressure SD	No reported isolation			Different to nutrient superdormancy	Wei et al., 2010

*Unless stated otherwise, heat activation was applied prior to nutrient germination for different species: B. subtilis: 75°C, 30 min; B. megaterium: 60°C, 15 min; B. cereus: 65°C, 20 min. ^1^Spores were heat activated at 70°C for 30 min. SD spore percentages were calculated based on observation of >440 spore for listed cases; ^2^LB medium: Luria-Bertani medium; ^3^AGFK (12 mM L-asparagine, 13 mM D-glucose, 13 mM D-fructose, 12.5 mM KPO_4_ buffer [pH 7.4]); ^4^AGFK (10 mM L-asparagine, 10 mM D-glucose, 10 mM D-fructose, 10 mM KCl in 25 mM KPO_4_ buffer [pH 7.4]).*

### Nutrient-SD Spores

The frequency of nutrient-SD spores among the total spore population generally varies between 1 and 12% with rich germinants (Ghosh and Setlow, 2009, 2010; Ghosh et al., 2009, 2012). Better germination conditions and heat activation result in a lower abundance of nutrient-SD spores (Ghosh and Setlow, 2009, 2010). However, the influence of heat activation was less significant when the spores were germinated in a nutrient-rich environment or with a mixture of nutrients that trigger multiple germination receptors (GRs) (Ghosh et al., 2009; Ghosh and Setlow, 2010). Generally, factors that influence the germination capacity of bacterial spores also affect the SD spore amount. These factors include stimulus type and intensity, heat activation, water activity, and so on (Setlow, 2003, 2014; Zhang et al., 2010; Abee et al., 2011; Lovdal et al., 2011; Christie, 2012).

The germination capacities of isolated nutrient-SD spores to different nutrient stimuli were intensively investigated (Ghosh and Setlow, 2009, 2010; Ghosh et al., 2009, 2012; Zhang et al., 2012). It was found that nutrient-SD spores require a heat activation temperature around 8–15°C higher than the initial dormant population (Ghosh et al., 2009). Nutrient-SD spores germinated poorly with the germinants that were originally used in their isolation and are more sensitive to a decrease in germinant concentration (Ghosh and Setlow, 2009; Ghosh et al., 2009; Zhang et al., 2012). A high-concentration mixture of nutrients increased the germination of nutrient-SD spores, but their germination efficiency and speed were still not as good as the initial dormant spores (Ghosh and Setlow, 2009; Ghosh et al., 2009). The germination of nutrient-SD spores with nutrients targeted to other GRs shows various behaviors. Some research has shown that they still germinate poorly (Ghosh and Setlow, 2009, 2010; Ghosh et al., 2009; Wei et al., 2010). Another research has indicated their germination was better as compared with the nutrients that were used to isolate them, but still worse than the initial dormant spores (Ghosh et al., 2012). Other authors have stated that nutrient-SD spores exposed to nutrients targeted to other GRs germinated almost as well as the initial dormant population (Zhang et al., 2012), or even more rapidly (Chen et al., 2014). The cause for the differences is unclear. Possibly due to slight differences in sporulation, germination, and isolation conditions, which could lead to differences on SD spore properties.

Although nutrient-SD spores germinate poorly with nutrient germinants, they germinate normally with Ca^2+^-DPA and dodecylamine (Ghosh and Setlow, 2009, 2010; Zhang et al., 2012). They were also reported to germinate similarly to the initial dormant population with bryostatin and purified peptidoglycan fragments (Wei et al., 2010). Moreover, it seems they can germinate as well as the initial spore population under HP treatment at both 150 MPa (37°C) and 500 MPa (50°C) (Wei et al., 2010). This is surprising, as it is generally considered that *Bacillus* spores germinate at 150 MPa via nutrient germination pathways. The discussed experimental results indicate that the cause of nutrient superdormancy is not the same as HP superdormancy. On the other hand, the isolation steps could have influenced the properties of SD spores. As reported by Chen et al. (2014) previously, some differences in protein levels between dormant and SD spores are similar to the protein changes during germination. This suggest that although SD spores were not committed to germination, small changes already took place in a non-committal way during the exposure to the nutrient germinants. These changes might be the cause that they could germinate normally under HP treatment, but not with the nutrient(s) that was used to isolate them.

Concerning the germination speed, nutrient-SD spores had a much longer individual lag time (*T*_lag_, which is the mean time between the spores coming into contact with nutrient germinants and the start of Ca^2+^-DPA release) (Zhang et al., 2012). Factors that influence the *T*_lag_ correlate with the factors that influence the SD spore level (Zhang et al., 2010), indicating that the *T*_lag_ represents the main cause of differences in germination speed between SD spores and the dormant spore population.

### Ca^2+^DPA-SD and Dodecylamine-SD Spores

Most SD spore studies have focused on nutrient SD spores, and only a limited amount of research has targeted populations that are reluctant to germination under the triggers of Ca^2+^-DPA and dodecylamine. The amounts of the Ca^2+^DPA-SD spores and dodecylamine-SD spores of *B. subtilis* are much lower than nutrient-SD spores. The amount of Ca^2+^DPA-SD spores is around 0.9% and dodecylamine-SD spores is around 0.4%, whereas that of nutrient SD spores is around 1–12% (Ghosh and Setlow, 2009; Perez-Valdespino et al., 2013). Research has revealed Ca^2+^DPA-SD spores germinate well with nutrient germinants and dodecylamine but poorly with Ca^2+^-DPA, while dodecylamine-SD spores germinate as well as the initial dormant population with nutrients and dodecylamine but germinate more slowly with Ca^2+^-DPA.

### High Pressure Superdormant Spores

High pressure processing inactivates bacterial spores by triggering relevant germination mechanisms. Notably, different HP treatments could induce the germination process, but the germination might be blocked in the intermediate phases and cannot be completed. However, as long as the relevant resistances of the spores are lost or significantly reduced, the following inactivation step could still inactivate them. Current state of art HP treatments alone cannot induce 100% germination (Mathys, 2008; Knorr et al., 2010; Reineke, 2012; Georget et al., 2014c,d; Dong et al., 2015; Georget, 2015; Sevenich and Mathys, 2018). The percentage of SD spores that remain dormant after HP treatment highly depends on the treatment conditions, including pH, water activity, pressure level, temperature, and dwell time (Mills et al., 1998; Wuytack et al., 1998; Considine et al., 2008; Reineke et al., 2013; Georget et al., 2014b; Bolumar et al., 2015; Rao et al., 2018). For example, decrease in water activities largely inhibits the germination of *B. cereus* spores by HP treatment (Al-Holy et al., 2007; Rao et al., 2018). The remaining SD spores are resistant and can survive HP treatment, thus limiting the application of HP processing as a milder non-thermal spore inactivation strategy. However, to our knowledge, there have been no reports of isolation and characterization of HPSD spores so far.

### Superdormant Spores of *Clostridium* Species

Spores of *Clostridium* species exhibit a similar germination heterogeneity like *Bacillus* species and *Clostridium* SD spores also occur (Webb et al., 2007; Rodriguez-Palacios and LeJeune, 2011; Stringer et al., 2011; Wang et al., 2011, 2012; Nerandzic and Donskey, 2013; Deng et al., 2015, 2017; Olguin-Araneda et al., 2015; Doona et al., 2016a). Similar to *Bacillus* species, the amount of *Clostridium* SD spores also depends on the factors that influence the germination efficiency. These influencing factors include heat activation, stimulus type and intensity (Wheeldon et al., 2008; Wang et al., 2011; Doona et al., 2016a). However, although the germination of *Clostridium* species and *Bacillus* species share some similarities, they also have a number of differences (Paredes-Sabja et al., 2011; Xiao et al., 2011; Christie, 2012; Brunt et al., 2014; Setlow, 2014; Setlow et al., 2017). For example, heat activation generally decreases the amount of nutrient-SD spores in *Bacillus*. In comparison, the effect of heat activation is more complex for *Clostridium* species (Ghosh and Setlow, 2009, 2010; Luu et al., 2015). The effect seems to be dependent on the germination/plating media (Montville, 1981), and on species, e.g., heat activation could stimulate the germination of *Clostridium perfringens* but not of several *Clostridium difficile* strains (Wang et al., 2011, 2015; Dembek et al., 2013; Doona et al., 2016a). The differences in germination between *Clostridium* and *Bacillus* species might indicate that their mechanisms of spore superdormancy are different. However, there has been much less work focusing on SD spores in *Clostridium* species than *Bacillus* species and there has been no report of the isolation of *Clostridium* SD spores.

## Potential Mechanisms of Spore Superdormancy

Superdormancy has been suggested to be an extreme form of germination heterogeneity and a strategy to ensure the survival of the entire population in a fast-changing environment (Veening et al., 2008; Ghosh and Setlow, 2010; Ghosh et al., 2012; Dembek et al., 2013). Obtaining a better understanding of spore superdormancy and its underlying mechanisms is crucial for the development of spore control strategies that are based on the germination-inactivation principle. Therefore, several research groups are currently investigating the genotypic and phenotypic differences between SD spores and their dormant counterparts. Currently, the exact causes of spore superdormancy are unclear and there is no consistent conclusion on whether the superdormancy of isolated SD spores is stable (Keynan et al., 1964; Ghosh and Setlow, 2010; Zhang et al., 2012). Previous research reported that nutrient-SD spores stored at −20°C for several months or even years could germinate similarly well compared to freshly isolated ones (Zhang et al., 2012), indicating the superdormancy could be permanent or at least stable for long time. However, another study reported that the isolated nutrient-SD spores stored at 4°C slowly lost their superdormancy. Even when they were stored at −20°C or −80°C, their germination ability still increased, but the rate of increase was significantly slower. Notably, although the germination capacity of nutrient-SD spores increased during cold storage, it did not reach the level of the initial dormant spore population (Ghosh and Setlow, 2010). Nevertheless, this indicates that the superdormancy of isolated SD spores is not permanent and it decreases over time.

Based on their findings, Ghosh and Setlow (2010) proposed that there are probably at least two causative factors for spore nutrient superdormancy, one permanent and one transient (Ghosh and Setlow, 2010). For the transient cause, Ghosh and Setlow (2010) suggested it might be related to the activation status of the spores, since heat activation, which is reversible, influences the frequency of nutrient-SD spores (Ghosh and Setlow, 2010). For the permanent cause, research has revealed that it is not because of genetic changes, since re-sporulated nutrient-SD spores showed the same germination capacity as the initial dormant population (Ghosh and Setlow, 2009; Chen et al., 2014). It was suggested that the phenotypic heterogeneity in germination may correspond to the presence of lower GR levels in the nutrient-SD spores (Ghosh and Setlow, 2009, 2010; Wei et al., 2010).

Lower GR levels as a cause for spore nutrient superdormancy has been proposed in many studies (Ghosh and Setlow, 2009, 2010; Ghosh et al., 2012). For example, in the study of Ghosh and Setlow (2009), the frequency of SD spores decreased dramatically when the level of GerB receptor increased. In their later research (Ghosh et al., 2012), they found that the level of GRs in SD spores was 6–10 fold lower than that in the initial dormant spores. Moreover, Chen et al. (2014) also found significant lower abundance of GerAC, GerKC, and GerD for *B. subtilis* nutrient-SD spores and proposed that a deficiency of GerD could be a reason for spore nutrient superdormancy. Lower GR levels as a causative factor of spore nutrient superdormancy is also supported by other evidence. First, the average amount of GRs per spore is low, thus, stochastic variation in the number could lead to the situation that a small proportion of spores have very few GRs and would probably germinate more slowly (Paidhungat and Setlow, 2001; Cabrera-Martinez et al., 2003; Setlow et al., 2012). Second, heat activation, which improved GR-mediated germination, can decrease the frequency of nutrient-SD spores (Ghosh and Setlow, 2009). Third, nutrient-SD spores germinate normally with Ca^2+^-DPA and dodecylamine, which both trigger spore germination through mechanisms that do not involve GRs (Paidhungat et al., 2001).

However, a lower number of GRs does not seem to explain the existence of other types of SD spores. For example, Ca^2+^DPA-SD spores were reported to have higher levels of GRs compared to the initial spore population. Their superdormancy could be due to lower levels of CwlJ, which is one of the cortex-lytic enzymes, and coat deficiency (Perez-Valdespino et al., 2013). Moreover, previous research suggested that the cause of HP superdormancy is different from that of nutrient superdormancy, since nutrient-SD spores can germinate normally with HP treatment (Wei et al., 2010).

Furthermore, *Bacillus* nutrient-SD spores showed a lower spore core water content than their dormant counterparts (Ghosh et al., 2009). This finding is consistent with the observation that spores sporulated at a higher temperature, which leads to a lower water content of the spore core (Melly et al., 2002), germinated less well than spores sporulated at a lower temperature (Gounina-Allouane et al., 2008; Markland, 2011; Markland et al., 2013b). This might indicate that a lower spore core water content could also be a cause of spore nutrient superdormancy (Cowan et al., 2003; Sunde et al., 2009; Setlow et al., 2012). One of the factors that leads to a difference in spore core water content is the DPA content of the spore. Although the DPA content of nutrient-SD spores is identical to that of initial dormant spores (Ghosh and Setlow, 2009), the environment of the DPA was found to be different, since the Raman spectral peaks of spore DPA differed between dormant and SD spores (Ghosh et al., 2009).

## Future Research Needs

So far, several types of SD spores have been characterized and mechanisms have been proposed for their superdormancy. However, the state of knowledge about some types of SD spores is still rudimentary and the exact mechanisms are not fully clear. Therefore, further research is needed to better understand SD spores, which represent one of the biggest challenges to the application of germination-inactivation as a milder non-thermal spore control strategy.

First, attention should be paid to HPSD spores in future research. To our knowledge, there have been no reports of the isolation and characterization of HPSD spores so far. This is somewhat surprising because from the applied perspective, there are advantages to triggering germination by HP rather than by nutrient/chemical stimuli. For example, HP can be used to evenly treat the final packed products without raising a risk of recontamination, while nutrient/chemical germination triggers need to be added and distributed into the foods. Moreover, HP triggers germination more homogeneously, while added nutrients or chemicals might have an inhomogeneous distribution, especially in solid foods, leading to inconsistent germination within the products. Furthermore, HP treatments can simultaneously trigger germination and inactivate the germinated spores, while spores germinated under nutrient/chemical triggers require further inactivation steps (Gould and Sale, 1970; Knorr et al., 1998, 2010; Georget et al., 2014a,c; Sevenich and Mathys, 2018). Additionally, previous research has suggested that the cause of spore HP superdormancy is different from spore nutrient superdormancy (Wei et al., 2010). Therefore, it would be beneficial to isolate and characterize HPSD spores regarding the mechanisms of their superdormancy. Such research would strongly support the implementation of milder HP-based spore control strategies.

Second, more attention should be paid to SD spores of *Clostridium* species, which have been far less studied than the SD spores of *Bacillus* species (Rodriguez-Palacios and LeJeune, 2011; Wang et al., 2011; Crowther et al., 2014; Deng et al., 2017). Since germination behavior varies among bacterial genera, further research is needed to clarify the properties of *Clostridium* SD spores and the underlying mechanisms of their superdormancy (Rodriguez-Palacios and LeJeune, 2011; Xiao et al., 2011; Deng et al., 2015).

Third, improvement of enumeration and culturing methods would be beneficial. Classic plate count methods based on quantifying colony-forming units are widely used to assess the viability of microbes. However, the number of colony-forming units is a measure of the highest physiological fitness of microbes (Bunthof, 2002), which might not be the best indicator for SD spores, because the possibility that these spores would not germinate on culture plates might lead to a risk of underestimation their numbers (Wells-Bennik et al., 2016). Therefore, tools such as flow cytometry or phase-contrast microscopy should be used to facilitate the enumeration of SD spores in future research. On the other hand, the amounts of SD spores are largely dependent on the germination conditions. Therefore, efforts should be put on improving the culturing methods to increase the recovery/germination of the SD spores. This is important for the accuracy of antimicrobial susceptibility tests, sterilization controls, and challenge tests (Silvestri et al., 2015; Wells-Bennik et al., 2016; Pereira and Sant’Ana, 2018).

Fourth, in order to successfully apply a germination-inactivation technology as a gentle safety control, several other aspects need to be considered besides spore germination. For example, the timing to apply the inactivation step is crucial. On one hand, it should be applied after the majority of spores lost most of their resistance. Spores should have enough time to pass germination stage II or at least to lose most of the Ca^2+^-DPA and reach a sufficient core hydration before a following inactivation step is considered (Moir et al., 1994; Setlow, 2003; Luu and Setlow, 2014). This time can vary, depending on spore species, germination stimuli and intensities. Notably, not all spores would finalize all their germination steps under a certain trigger (Wuytack et al., 1998; Reineke, 2012), but as long as a relevant spore resistance is lost, they could be efficiently inactivated by a gentle inactivation step.

On the other hand, the germination-inactivation approach focuses on the elimination of bacterial spores to ensure the microbiological safety of the products, but the absence of spores does not guarantee the absence of toxins. Some pathogenic spore-forming bacteria can produce toxins, which could endanger consumers. Different situations need to be taken into account if the germination-inactivation approach is considered as a food safety control in this case. First, special focus needs to be put on spore species that can produce toxins during the growth phase after their germination. For example, *B. cereus* can produce diarrheagenic or emetic toxins during the exponential or the stationary phase of growth respectively (Roberts and Tompkin, 1996; Brown, 2000; Ceuppens et al., 2012), while *Clostridium botulinum* and *C. difficile* synthesize toxins in the late exponential growth phase and beginning of the stationary phase (Voth and Ballard, 2005; Proft, 2009). It is essential to consider the germination velocity rates and control the time intervals between the germination and inactivation steps to ensure food safety for these cases. Notably, in any case, an inactivation needs to be performed before germinated spores could sporulate again. The time needed to complete sporulation varies, and it takes approximately 8–10 h in *B. subtilis* (Robleto et al., 2012). Proper processing time windows need to be identified using predictive models and experimental validation tests to ensure that the inactivation step is performed in the specific time period where the majority of spores lost most of their resistances but did not start producing toxins or sporulation, yet.

Another situation is where toxins are already present in the product, either produced by vegetative cells in their late growth phases or during sporulation, e.g., *C. perfringens* produces heat sensitive enterotoxin during sporulation and releases the toxin when the mother cell lysis (Duncan et al., 1972; Uemura, 1978). In this case, the following inactivation step needs to be able to degrade the present toxins, e.g., for heat sensitive toxins a mild heat inactivation step could be applied. For heat stable toxins, e.g., *B. cereus* emetic toxin, a mild heat step after germination might remove the sensitized spores but not the toxins. In this case, other approaches to control the toxin levels are needed. Generally, it is important to control the quality of raw material inputs, ingredients and their storage conditions to prevent the toxin formation before germination-inactivation steps.

Finally, knowledge obtained from SD spore research could be used to develop milder spore control strategies. On one hand, germination-inactivation technologies by first triggering spore germination and followed by a gentle inactivation step to inactivate the sensitized spores could be further developed and improved. Spore germination could be maximized when we understand the mechanisms and the influencing factors for spore superdormancy. For example, germination percentages can be increased by combining various germination triggers or controlling the influencing factors. Important influencing factors include heat activation, germination stimulus type and intensity (Wei et al., 2010; Lovdal et al., 2011). Besides that, from the application point of view, it is important to understand the germination behavior of spores that are formed and present in the food products. This is especially relevant as the sporulation conditions, which influence the spore germination properties, are often unknown and not controlled in this case (Wells-Bennik et al., 2016). Moreover, spores germination behaviors might be completely different when spores are germinated in food matrices compared to buffer systems. For example, the germination of *Bacillus* spores by nutrient and HP were inhibited when they are present in foods with low water activity (Al-Holy et al., 2007; Rao et al., 2018). Therefore, future research is needed to investigate the mechanisms of how different factors influence spore germination. On the other hand, since only germinated spores proliferate and cause problems, hurdles can be put in place to inhibit the germination/outgrowth of the remaining SD spores. Examples of these hurdles can be pH, temperature, or bacteriocins such as nisin (Markland et al., 2013a; Nerandzic and Donskey, 2013; Patrignani and Lanciotti, 2016; Wells-Bennik et al., 2016).

## Conclusion

Research on SD spores will help reveal factors that contribute to their superdormancy and allow for the identification of the underlying mechanisms that lead to their extremely low germination capacity as compared to the whole population. It will also contribute to improved predictive models that take germination heterogeneity into account, which can provide a mechanistic understanding of spore germination processes. Additionally, it will provide a foundation for developing milder non-thermal spore control strategies based on the germination-inactivation principle. This could help to ensure microbial safety and quality retention of food products, contributing significantly to providing fresher and more nutritional foods for consumers. Moreover, aside from the food sector, the medical, pharmaceutical, and (bio)chemical sectors, where spore eradication is needed, will also benefit from research on SD spores, especially for the sterilization of heat-sensitive products.

## Author Contributions

YZ and AM contributed to the manuscript at all stages.

## Conflict of Interest Statement

The authors declare that the research was conducted in the absence of any commercial or financial relationships that could be construed as a potential conflict of interest.
